# Supplementary material for: Describing fine spatiotemporal dynamics of rat fleas in an insular ecosystem enlightens abiotic drivers of murine typhus incidence in humans
Source: PLoS Negl Trop Dis. 2021 Feb 18;15(2):e0009029. doi: 10.1371/journal.pntd.0009029 (PMC7924756; doi:10.1371/journal.pntd.0009029)
Supplement: S1 Table — (PDF) [file pntd.0009029.s001.pdf]

Describing fine spatiotemporal dynamics of rat fleas in an insular ecosystem enlightens abiotic drivers of murine typhus incidence in humans

Annelise Tran, Gildas Le Minter, Elsa Balleydier, Anaïs Etheves, Morgane Laval, Floriane Boucher, Vanina Guernier, Erwan Lagadec, Patrick Mavingui, Eric Cardinale, Pablo Tortosa

Supporting information

S1 Table. Location of the study sites

Dataset	Site	Longitude (WGS84)	Latitude (WGS84)
1	BRAS DES CALUMETS	55.606128	-21.148718
1	CHEMIN FEOGA	55.324363	-21.029138
1	COL DE BELLEVUE	55.590013	-21.165949
1	ETANG ST PAUL 1	55.298071	-20.988898
1	ETANG ST PAUL 2	55.302499	-20.991364
1	FORET TAMARINS MAIDO	55.367195	-21.060532
1	GRAND ETANG	55.645291	-21.095118
1	GRAND FOND TAKAMAKA 1	55.630139	-21.076289
1	GRAND FOND TAKAMAKA 2	55.634250	-21.070641
1	ILET COCO	55.689739	-21.042165
1	MAIDO	55.388447	-21.075686
1	PIC ADAM	55.467778	-20.937539
1	PLAINE DES CAFRES	55.551465	-21.172565

1	PLAINE DES PALMISTES	55.657375	-21.110117
1	PLANTATION MELISSA	55.689088	-21.045910
1	PORT EST 1	55.329556	-20.931738
1	PORT EST 2	55.320376	-20.937485
1	PORT EST 3	55.315637	-20.934419
1	PORT EST 4	55.309273	-20.931089
1	RAVINE 3 BASSINS 1	55.259558	-21.109934
1	RAVINE 3 BASSINS 2	55.257115	-21.111374
1	RF BOIS DE NEFLES	55.359769	-21.023143
1	RIVIERE DES PLUIES 1	55.501817	-20.934212
1	RIVIERE DES PLUIES 2	55.500214	-20.943693
1	RIVIERE DES PLUIES 3	55.495081	-20.951922
1	RIVIERE DES ROCHES FRONT DE MER	55.701324	-21.013471
1	SANS SOUCIS 2	55.320959	-20.973241
1	SAVANE ST LEU 1	55.290259	-21.205031
1	SAVANE ST LEU 2	55.289546	-21.196393
2	GILLOT	55.515954	-20.895446
2	RAVINE A MARQUET	55.329579	-20.93131
2	PORT EST	55.321001	-20.936132
2	RIVIERE DES GALETS	55.32445	-20.968024
2	PIERREFOND	55.426876	-21.318233
2	BOIS D’OLIVE	55.444062	-21.298439
