# Supplementary material for: Describing fine spatiotemporal dynamics of rat fleas in an insular ecosystem enlightens abiotic drivers of murine typhus incidence in humans
Source: PLoS Negl Trop Dis. 2021 Feb 18;15(2):e0009029. doi: 10.1371/journal.pntd.0009029 (PMC7924756; doi:10.1371/journal.pntd.0009029)
Supplement: S2 Table — (PDF) [file pntd.0009029.s002.pdf]

Describing fine spatiotemporal dynamics of rat fleas in an insular ecosystem enlightens abiotic drivers of murine typhus incidence in humans

Annelise Tran, Gildas Le Minter, Elsa Balleydier, Anaïs Etheves, Morgane Laval, Floriane Boucher, Vanina Guernier, Erwan Lagadec, Patrick Mavingui, Eric Cardinale, Pablo Tortosa

Supporting information

S2 Table. Dataset 1

SITE	MAMMALS	XENOPSYLLA	GFI
BRAS DES CALUMETS	21	0	0.00
CHEMIN FEOGA	29	2	0.07
COL DE BELLEVUE	19	0	0.00
ETANG ST PAUL 1	18	3	0.17
ETANG ST PAUL 2	26	9	0.35
FORET TAMARINS MAIDO	29	0	0.00
GRAND ETANG	28	0	0.00
GRAND FOND TAKAMAKA 1	13	0	0.00
GRAND FOND TAKAMAKA 2	12	0	0.00
ILET COCO	10	0	0.00
MAIDO	29	16	0.55
PIC ADAM	24	1	0.04
PLAINE DES CAFRES	38	2	0.05
PLAINE DES PALMISTES	61	0	0.00
PLANTATION MELISSA	30	0	0.00
PORT EST 1	15	0	0.00
PORT EST 2	25	15	0.60
PORT EST 3	6	1	0.17
PORT EST 4	1	0	0.00
RAVINE 3 BASSINS 1	21	3	0.14
RAVINE 3 BASSINS 2	5	19	3.80
RF BOIS DE NEFLES	29	0	0.00
RIVIERE DES PLUIES 1	10	1	0.10
RIVIERE DES PLUIES 2	12	0	0.00
RIVIERE DES PLUIES 3	6	1	0.17
RIVIERE DES ROCHES FRONT DE MER	35	0	0.00
SANS SOUCIS 2	31	48	1.55
SAVANE ST LEU 1	13	21	1.62
SAVANE ST LEU 2	3	33	11.00
