# Supplementary material for: Describing fine spatiotemporal dynamics of rat fleas in an insular ecosystem enlightens abiotic drivers of murine typhus incidence in humans
Source: PLoS Negl Trop Dis. 2021 Feb 18;15(2):e0009029. doi: 10.1371/journal.pntd.0009029 (PMC7924756; doi:10.1371/journal.pntd.0009029)
Supplement: S3 Table — (PDF) [file pntd.0009029.s003.pdf]

Describing fine spatiotemporal dynamics of rat fleas in an insular ecosystem enlightens abiotic drivers of murine typhus incidence in humans

Annelise Tran, Gildas Le Minter, Elsa Balleydier, Anaïs Etheves, Morgane Laval, Floriane Boucher, Vanina Guernier, Erwan Lagadec, Patrick Mavingui, Eric Cardinale, Pablo Tortosa

Supporting information

S3 Table. Dataset 2

Site	Date	Rodents	Xenopsylla	GFI
Gillot	01/03/2017	6	0	0.00
Gillot	23/05/2017	8	0	0.00
Gillot	17/08/2017	5	0	0.00
Gillot	06/11/2017	4	0	0.00
Gillot	20/09/2017	19	0	0.00
Gillot	08/01/2018	15	2	0.13
Ravine a Marquet	06/03/2017	40	4	0.10
Ravine a Marquet	16/05/2017	34	0	0.00
Ravine a Marquet	17/07/2017	18	0	0.00
Ravine a Marquet	05/12/2017	26	0	0.00
Ravine a Marquet	05/02/2018	46	2	0.04
Port Est	10/04/2017	23	3	0.14
Port Est	29/05/2017	25	5	0.20
Port Est	29/08/2017	20	21	1.11
Port Est	11/12/2017	22	21	0.95
Port Est	12/02/2018	22	14	0.64
Pierrefond	04/04/2017	8	6	1.00
Pierrefond	13/06/2017	23	31	1.35
Pierrefond	21/08/2017	22	18	0.82
Pierrefond	21/10/2017	19	188	8.95
Pierrefond	31/01/2018	20	10	0.56
Riviere des Galets	18/04/2017	17	9	0.53
Riviere des Galets	19/06/2017	26	12	0.46
Riviere des Galets	05/09/2017	14	21	1.50
Bois d'Olive	06/06/2017	20	14	0.74
Bois d'Olive	12/09/2017	21	77	3.85
Bois d'Olive	19/12/2017	12	36	3.00
Bois d'Olive	20/02/2018	29	2	0.07
