# Supplementary material for: Describing fine spatiotemporal dynamics of rat fleas in an insular ecosystem enlightens abiotic drivers of murine typhus incidence in humans
Source: PLoS Negl Trop Dis. 2021 Feb 18;15(2):e0009029. doi: 10.1371/journal.pntd.0009029 (PMC7924756; doi:10.1371/journal.pntd.0009029)
Supplement: S4 Table — (PDF) [file pntd.0009029.s004.pdf]

Describing fine spatiotemporal dynamics of rat fleas in an insular ecosystem enlightens abiotic drivers of murine typhus incidence in humans

Annelise Tran, Gildas Le Minter, Elsa Balleydier, Anaïs Etheves, Morgane Laval, Floriane Boucher, Vanina Guernier, Erwan Lagadec, Patrick Mavingui, Eric Cardinale, Pablo Tortosa

Supporting information

S4 Table. Results of univariate analysis

Variable	Estimate	p-value
**Annual median rainfall (mm)**	**$-2.687\ 10^{-3}$**	***
**Annual minimum temperature (°C)**	**0.335**	***
**Annual mean temperature (°C)**	**0.404**	***
**Annual maximum temperature (°C)**	**0.560**	***
Continuous urban	-131.843	NS
**Discontinuous urban**	**7.019**	***
**Forest**	**-6.224**	***
**Shrub vegetation**	**2.101**	***
**Herbaceous vegetation**	**6.463**	***
**Barren land**	**3.309**	***
Water	$-9.706\ 10^{3}$	NS
**Agriculture**	**-2.248**	***

*** p<0.001 ** p<0.01 * p<0.05 . p<0.1 NS : non significant
