# Supplementary material for: Describing fine spatiotemporal dynamics of rat fleas in an insular ecosystem enlightens abiotic drivers of murine typhus incidence in humans
Source: PLoS Negl Trop Dis. 2021 Feb 18;15(2):e0009029. doi: 10.1371/journal.pntd.0009029 (PMC7924756; doi:10.1371/journal.pntd.0009029)
Supplement: S5 Table — TMAX_N denotes the maximum temperature over the last N days, TMIN_N denotes the minimum temperature over the last N days, RAINMAX_N denotes the maximum rainfall over the last N days, and RAIN_N denotes the rain accumulation over the last N days. Models are ordered from best to worst. (PDF) [file pntd.0009029.s005.pdf]

Describing fine spatiotemporal dynamics of rat fleas in an insular ecosystem enlightens abiotic drivers of murine typhus incidence in humans

Annelise Tran, Gildas Le Minter, Elsa Balleydier, Anaïs Etheves, Morgane Laval, Floriane Boucher, Vanina Guernier, Erwan Lagadec, Patrick Mavingui, Eric Cardinale, Pablo Tortosa

Supporting information

S5 Table. Results of SVM analysis: list of the ten models with the best performance in terms of mean square error. *TMAX_N* denotes the maximum temperature over the last *N* days, *TMIN_N* denotes the minimum temperature over the last *N* days, *RAINMAX_N* denotes the maximum rainfall over the last *N* days, and *RAIN_N* denotes the rain accumulation over the last *N* days. Models are ordered from best to worst.

Model	Input 1	Input2	Mean square error
1	TMAX_0	RAINMAX_42	1.753
2	TMAX_0	RAINMAX_49	1.753
3	TMIN_0	RAIN_56	1.805
4	RAINMAX_42		1.819
5	RAINMAX_49		1.819
6	TMIN_0	RAINMAX_42	1.825
7	TMIN_0	RAINMAX_49	1.825
8	TMIN_0	RAIN_63	1.825
9	TMAX_0	RAIN_49	1.852
10	TMAX_0	RAIN_42	1.873
