# Supplementary material for: Describing fine spatiotemporal dynamics of rat fleas in an insular ecosystem enlightens abiotic drivers of murine typhus incidence in humans
Source: PLoS Negl Trop Dis. 2021 Feb 18;15(2):e0009029. doi: 10.1371/journal.pntd.0009029 (PMC7924756; doi:10.1371/journal.pntd.0009029)
Supplement: S1 Text — (PDF) [file pntd.0009029.s006.pdf]

Describing fine spatiotemporal dynamics of rat fleas in an insular ecosystem enlightens abiotic drivers of murine typhus incidence in humans

Annelise Tran, Gildas Le Minter, Elsa Balleydier, Anaïs Etheves, Morgane Laval, Floriane Boucher, Vanina Guernier, Erwan Lagadec, Patrick Mavingui, Eric Cardinale, Pablo Tortosa

Supporting information

S1 file. Trapping protocol, Dataset 2

A longitudinal trapping survey was conducted in six study sites (Fig 1, Tables S1 and S3) between March 2017 and February 2018. Small mammal trapping was conducted following a standardized protocol: on each sampling site, 50 to 100 peanut butter baited live traps were placed in line approximatively 10 meters apart in the afternoon; trapped animals were collected the following morning and brought to the laboratory. Animals were euthanized by cervical dislocation and ectoparasites immediately collected and preserved in 70% ethanol. *Rattus* spp. was identified using morphological criteria including the comparison of the ratio of tail to body lengths, the ear length and the hind foot length [1]. Morphological diagnosis of fleas was realized using a taxonomic key provided by Anne Laudisoit (pers. com.).

1. Pages M, Chaval Y, Herbreteau V, Waengsothorn S, Cosson JF, Hugot JP, et al. Revisiting the taxonomy of the Rattini tribe: a phylogeny-based delimitation of species boundaries. BMC Evol Biol. 2010; 10:184. doi: 10.1186/1471-2148-10-184
