# Supplementary material for: Describing fine spatiotemporal dynamics of rat fleas in an insular ecosystem enlightens abiotic drivers of murine typhus incidence in humans
Source: PLoS Negl Trop Dis. 2021 Feb 18;15(2):e0009029. doi: 10.1371/journal.pntd.0009029 (PMC7924756; doi:10.1371/journal.pntd.0009029)
Supplement: Attachment — Submitted filename: Response_Reviewers.docx [file pntd.0009029.s007.docx]

PNTD-D-20-01203

Describing fine spatiotemporal dynamics of rat fleas in an insular ecosystem enlightens abiotic drivers of murine typhus incidence in humans

Rebuttal letter

**Dear Editor and Referees,**

Thank you for your very careful review of our paper, and for the positive comments, corrections and suggestions that ensued. The paper was revised to take all of them into account.

In the present letter, we sequentially address all of the points raised by each reviewer (answers to comments or requests are in blue in the text).

Reviewer #1: I found the choice of words odd and awkward in a few places, and specific suggestions for more appropriate word choices and sentence structure are listed below.

Specific suggestions:

Line 27: change structuration to structure, delete "incident" before cases

Done.

Line 29: change "compartment" to "component"

Done.

Line 30: change "of" to "from"

Done.

Line 49; awkward sentence: change “which number of cases” to “as the number of human cases”….

Done.

Line 52: delete "thus"

Done.

Line 67: change "highlight" to "determine"

Done.

Line 78: add comma after niche

Done.

Line 84: change "setups" to "settings"

Done.

Line 94: change "up-cited" to "previous"

Not applicable, as in the revised version of the paper, the introduction was shortened and the details on dengue and chikungunya emergences were removed, according to a comment of Reviewer2.

Line 94: Authors state “its emergence in Reunion Island may have

resulted from suboptimal surveillance and diagnosis prior the identification of the first

incident cases in 2012. This sentence needs to be reworded…e.g. detection of emergence may have been delayed due to suboptimal surveillance and diagnosis prior to identification of the first human cases in 2012.

The sentence was reworded as follows: “the detection of its emergence in Reunion Island may have been delayed due to suboptimal surveillance and diagnosis prior the identification of the first human cases in 2012”.

Line 97: disease should be diseases

The sentence was clarified: “diseases such as murine typhus”

Line 100: structuration is a strange word, replace with structure here and throughout the paper

Done.

Line 101: add "with" after seasonality, and before cases

Done.

Line 104: “it typically is” should be “as is typical”

Done.

Line 118: change "up-cited" to "previously stated"

Done.

Line 120: incident can be deleted…human cases is sufficient

Done.

Line 124: "aiming" should be "aimed"

Done.

Line 133; insert comma after surveillance, delete “an access to”

Done.

Line 134; insert “access” before “to health data”

Done.

Lines 135 and 136: replace “run” with “conducted according to”

Done.

Line 155: replace “a” with “rodent”

Done.

Line 169: add “in humans” after cases, reemphasize throughout that you are referring to incidence or cases of human typhus

Done.

Line 259: Univariable should be univariate here and elsewhere

Done.

Line 260: multivariable should be multivariate here and elsewhere

Done.

Line 320: replace “leading to” with “and a” (non specific symptoms don’t lead to lack of awareness but the two combined lead to underestimation)

We agree. Done.

Line 323: replace “explored rodents and rodent fleas compartments” with “ analyzed factors related to rodents and rodent fleas, ….

Done.

Line 324: replace “the presented analysis enlightens” with “ The results of our analysis demonstrates that climatic and environmental variables strongly affect….

Done.

Line 327: replace structuration with "occurrence"

Done.

Line 331: flea instead of fleas

Done.

Line 340: burrow instead of burrows before architecture

Done.

Line 341: delete "burrows"

Done.

Line 376: delete "somehow"

Done.

Line 377: delete set-ups. Change environment to environmentally.

Done.

Line 380: change “an exploration” to “studies”

Done.

Line 381: Not sure what pre-imaginal means, use off-host or questing fleas

Done.

Line 382: Not sure what after the “seasonal pick” means…please clarify

Sorry, this was a spelling error. We replaced by “the seasonal peak of abundance”.

Line 407: "theses" should be "these"

Corrected.

Line 437: set-up should be "setting"

Done.

Line 442: delete "this"

Done.

Reviewer #1: In general, this is an interesting and well-organized study on factors associated with rats and rat fleas and the incidence of human typhus on Reunion Island. The authors did a nice job describing the problem, their study design and their findings and relating that to broader human health implications.

Thank you very much for your comments and careful revision of the manuscript.

Reviewer #2: The manuscript by Tran and colleagues describes the environmental conditions that appear favorable to Xenopsylla infestation of rats on Reunion Island, where murine typhus has emerged as a cause of illness – especially in the dryer areas of the south and western parts of the island. On a local level (i.e., Reunion Island) the results can have an impact on control efforts. Of broader interest, the authors’ approach could help others postulate how to understand and approach factors associated with R. typhi transmission in other regions. Below are some minor suggestions and queries for the authors’ consideration.

Line 55: Suggest qualifying the phrase “main determinants” as of those investigated within the confines of this study.

Agreed. We modified the sentence into:

“Results showed that i) rat fleas’ abundance is highly dynamic, characterized by a peak at the end of the dry season and ii) among the factors investigated, rainfall, temperature and land cover are the main determinants of rat fleas’ abundance.”

Lines 87 – 94: The details regarding the emergence of chikungunya, dengue, and how they are different than that of R. typhi is not necessary for an already lengthy introduction.

The introduction was shortened as follows:

“Reunion Island, a geographically isolated volcanic island located 700 km East of Madagascar has experienced in the last decade the emergence of several vector borne diseases [4, 5], including murine typhus caused by the bacterium Rickettsia typhi [6]. The detection of the emergence of this disease in the island may have been delayed due to suboptimal surveillance and diagnosis prior the identification of the first human cases in 2012 [7].”

Line 97: Suggest finding an alternate phrase for “off the radar.”

“off the radar” was replaced by “under-reported”

Line 104: Provide citation(s) (additional to #10) to support that rats are dispersed in a widespread manner on tropical islands.

Done. We added the following references:

Drake, D. R., & Hunt, T. L. (2008). Invasive rodents on islands: Integrating historical and contemporary ecology. Biological Invasions, 11(7), 1483–1487. doi: 10.1007/s10530-008-9392-1

Harper, G. A., & Bunbury, N. (2015). Invasive rats on tropical islands: Their population biology and impacts on native species. Global Ecology and Conservation, 3, 607–627. doi: 10.1016/j.gecco.2015.02.010

Line 177 – 178: How is the serologic confirmation by the reference center different than the previously stated serologic criteria? If different, the methods or criteria should be stated. If the same, the reference center can be mentioned as a resource in the text but not a specific criterion in the bulleted list.

Thanks for this comment. The method used by the Rickettsia National Reference Center was clarified:

-	A positive serology confirmed by Western blot by the Rickettsia National Reference Center (Marseille, France)

Line 229: Very minor: The phrase “GFI index” seems redundant. Would just “GFI” suffice?

Agreed and corrected.

Line 297 – 298: Can the authors provide the length of lag time between rapid decrease in GFI and the eventual decrease in human cases? Although it might be expected that there might be a lag, this might be counter intuitive to some readers. The lag time length (if relatively short) might help put things into more plausible context.

Indeed, the lag time between GFI and number of human cases is about two months. We clarified it in the text.

“ […]predicted GFI rapidly decreases to reach its minimum in February, while the number of human cases remains high and drops with a time lag of two months after the GFI decrease.”

Lines 346 – 354: Can the authors’ compare and contrast the types of land cover classes here (those with impact versus those without impact) and offer hypotheses as to why they are different? Although some differences in regard to presence of reservoirs/vectors seem obvious (i.e., continuous urban and water), others are not (e.g., shrub vegetation versus herbaceous vegetation).

Thank you for stressing this point. In the multivariate analysis, no significant correlation (positive or negative) was found for the classes “continuous urban”, “shrub vegetation”, “barren land”, and “water”. However, significant and positive associations were found for the classes “shrub vegetation” and “barren land” in the univariate analysis, thus it seems not possible to conclude about the importance of these types of land cover classes. In the Discussion section, we stress the importance of the additional sampling and studies to better understand the role of landscape elements in rat fleas’ abundances.

“Of note, the land cover classes “continuous urban”, and “water” were both negatively correlated with rat flea abundance in the univariate analysis (Table S4). On the other hand, the land cover classes “shrub vegetation” and “barren land” were not retained in the multivariate model, although they were positively and significantly correlated with rat flea abundance in the univariate analysis (Table S4). These results stress the need for additional sampling studies to decipher the role of landscape elements in rat flea abundances in Reunion Island.”

Reviewer #3: (No Response)

--------------------
